# Supplementary material for: Are carbon emissions trading and green financial instruments synergistic? -Comprehensive quantitative research based on content analysis
Source: PLoS One. 2024 Mar 7;19(3):e0298601. doi: 10.1371/journal.pone.0298601 (PMC10919601; doi:10.1371/journal.pone.0298601)
Supplement: S1 Data — (ZIP) [file pone.0298601.s001.zip › Data/地级市Z_F工作报告.zip!/╕ú╜¿/─╧╞╜╩╨/└┤╫╘╣1⁄2╓┌║┼í╛┬φ┐╦╩2╛▌═°í┐_2009.docx]

连续三年来，我在一年一度的全市“两会”结束之后，都到党校和相关单位为大家解读政府工作报告。前两年，我着重从报告原文的工作重点、内容和方向进行分析。今年，我主要对报告引申出来的观念转变问题进行解读，希望为全市解放思想、转变观念、促进科学发展、服务跃升发展作一些贡献。

　　一、2009年政府工作报告的主要内容和特点

　　今年2月5日，龚清概市长在南平市三届人大四次会议上作了题为《科学发展 先行先试 加快推进海西绿色腹地建设》的政府工作报告。这份报告共23页1.3万字，是历年来比较短的报告，但涵盖的内容却比较广。在报告起草之前，去年11月份期间，龚市长要求对两年来推进跃升发展调研工作所取得的成效，进行收集汇总，并在报告中体现，形成一份好的报告统一思想、凝聚人心、推动发展。在龚市长的直接指导下，我们形成了这份言简意赅的报告。

　　报告主要围绕一条主线，即坚持科学发展，要求实现一个目标，即推进海西绿色腹地跃升发展。报告回答了三个问题：一是如何保持跃升发展势头？在国际金融危机的严峻形势下，我们能不能保持跃升发展、怎么保持跃升发展，报告用了比较大的篇幅进行论述。二是如何实现科学发展？在全党全国开展深入学习实践科学发展观活动中，我们将用什么样的发展方式引领闽北科学、跃升发展，报告也作了回答。三是如何抓住当前历史性机遇。面对复杂的经济环境，党中央、国务院出台了一系列的保增长、扩内需、调结构、重民生的政策措施，对闽北来说是难得的历史性机遇，我们如何抢抓，如何把握，如何用好，报告系统地提出了闽北加快发展的举措。为此，这份报告分为三个部分，第一部分，奋起争先、加快发展的一年。报告本着实事求是的原则，从八个方面总结了2008年政府的工作成效：跃升发展态势持续，产业发展水平提升，“三农”工作扎实推进，发展条件加快改善，城市建设力度加大，社会事业加快发展，惠民举措得到落实，和谐社会持续构建。第二部分，坚持科学发展，扎实做好2009年工作。第三部分，有效作为，提升领导科学发展能力。从积极应对挑战、转变政府职能、推进依法行政、坚持从严治政四个方面对政府自身建设提出了要求。报告经人大审议后的正式稿，已全文刊登在今年2月15日的《闽北日报》

　　从报告的内容和结构来看，体现了三个特点：

　　一、目标的坚定性。2008年我市发展速度开始赶上全省平均水平。在这种发展速度下，2009年的经济社会预期指标怎么安排？在讨论指标安排时，各方意见非常不一，有人认为作为后发展地区的发展速度必须快一点，以鼓舞人心；有人认为必须慎重，因为全球金融危机对后发展地区呈现出滞后效应，在2009年对闽北发展可能产生比较严重的影响。最后经过各方面的权衡，将GDP增幅定为13.2%，高于全省和三明、龙岩、宁德的指标安排。确定这个指标基于两个理由：一是按照“十一五”中期评估，今年我市GDP增长指标要达到14.1%－14.3%之间，才能实现三届人大三次会议调整之后“十一五”规划目标，所以后两年要快一点；二是考虑宏观环境复杂多变，必须留有余地，指标安排不能过高。可以说，13.2%的GDP增幅指标既权衡了发展基础和目标要求，也考虑到全省平均水平和周边地区的发展。GDP增长13.2%的指标确定之后，我们又系统考虑了规模工业、财政收入、固定资产投资、城乡居民收入等支撑指标，各县（市、区）和各部门也根据这个指标确定了各自的发展目标。对此，南平的广大干部必须坚定信心，主动作为，善于从变化的形势中捕捉和创造发展机遇，在不利环境中寻找和培育有利因素，坚定不移地推动闽北经济社会又好又快发展。

　　二、工作的持续性。党的十六大以来，省委作出构建海峡西岸经济区战略决策之后，南平市委、市政府着眼于融入全国全省大局、发挥闽北比较优势，确定了“建设开放、发展、和谐的海峡西岸经济区绿色腹地”的发展目标定位。2007年南平市新一届政府班子成立以来，按照党的十七大提出全面建设小康社会的新要求，进一步明确了海西绿色腹地跃升发展的目标任务，形成了严密的海西绿色腹地内涵，就是“构建海峡西岸的绿色腹地、构建南接北联的战略通道、构建连片发展的前锋平台”。这三个构建就是对海西绿色腹地定位之后的深化、拓展和延伸。今年的报告，我们按照科学发展观的要求，继续对跃升发展战略进行拓展与深化，对去年提出的“好”、“锋”、“通”、“民”、“和”五个字要求，进行详细而具体的部署，形成做好2009年工作的五个方面内容。

　　一是保持“好”的态势，实践科学发展。这一部分内容写出了南平自己的特色。报告提出，“充分发挥生态资源优势，突出发展生态主导型的区域经济，转变发展方式，优化经济结构，走高起点、集约化、可持续的发展道路，保持较快发展速度，实现闽北奋起争先、跃升发展”。其中关键字眼是“较快发展速度”，因为假如速度很慢，总量很小，“好”就无从谈起，奋起争先、跃升发展目标也不可能实现。报告对“保持‘好’的态势、实践科学发展”进行了具体化，提出要“持续突出突破工业、扎实推进农村发展、提升第三产业发展水平”。

　　二是发挥“锋”的作用，致力先行先试。省委在构建海西战略中，要求闽北发挥纵深推进的前锋作用，实现北联西进、延伸两翼、对接两洲，服务中西部，连片发展。闽北将由“沿海的山区”变“东部的沿海”，所以要致力先行先试。报告提出，“充分发挥空间资源优势，推进城市化先行，做大产业平台，构建对外开放载体，打造先行先试窗口，形成产业集聚基地，在海西建设中体现作为”。具体来说，要坚持城市化先行，为工业化提供条件；加速拓展工业平台，大力推进市本级和县市平台建设，为跃升发展提供载体；强化项目带动和品牌经营，创新体制机制，为推进发展提供工作方式方法。

　　三是做足“通”的文章，力争能快则快。闽北的落后，某种意义上说，就是交通设施的落后。南平开通高速公路的时间，比沿海地区晚十年，比周边地市晚五年，这可能是造成我们30年“河西”的一个重要原因。因此，能快则快地做足“通”的文章，实现时空转换，加快融入发达地区的经济半径。报告提出，“加速改善交通基础设施条件，构建现代立体交通体系，实现闽北与发达地区的时空转换，着力突破能源和资金瓶颈制约，加快融入发达地区的经济半径”。龚市长在多次会议上，向广大干部说了一个观点，1988年广东惠阳地区一分为四，分为东莞、深圳、惠州、汕头，并同时作为经济特区。几十年过去了，东莞、深圳发展起来了，但另两个变化不明显，未呈现跳跃式的发展。其原因是，按照经济半径理论，3个小时的车程，也就是能在一天之内坐车来回的区域，才能融入发达地方的经济半径。东莞、深圳离香港仅1.5—2小时的车程，时空转换比较到位，成功融入香港的经济发展半径。而惠州虽然一马平川，立地条件较好，但到香港要5个小时车程，自然无法融入香港的经济半径。汕头也是如此。反观闽北，在高速公路未通车时，武夷山到福州要5个小时车程，现在只要3个小时。

　　四是突出“民”的利益，注重实惠实效。报告提到，“坚持把新增财力更多地用于改善民生，统筹发展社会事业，提高教育、医疗、文化等公共服务水平，让改革发展成果更好地惠及人民，实现好、维护好、发展好人民群众的根本利益”。这一点大家都比较清楚，我们发展的目的就是为民。

　　五是营造“和”的氛围，重在凝心聚力。特别在当前经济危机面前，信心胜于黄金，民心胜于黄金。我们要一切为了企业发展，一切服务企业发展，调动各方积极因素，凝聚各方力量，共度难关，共克时妈。所以报告提出，“倍加珍惜、倍加爱护业已形成的风正气顺、人和业兴的良好氛围，妥善解决好各种矛盾和问题，统筹协调各方利益，最大限度地调动一切积极因素，形成跃升发展的强大合力”。

　　三、要求的约束性。今年报告的文风有几个少，说理论的少，抄上面的少，模糊概念的少，所以报告比较精简地对政府工作进行了部署。但实际上，我们根据报告开展工作又非常难，因为报告对每一项工作都提出了定性定量的约束性的要求。如报告提出，“加快沿江快速通道和景观通道建设，大力推进两批48项城建重点项目，实施中心城区进城通道和防洪堤等工程建设”。短短的几十个字，就有大量的工作要做。总之，报告的每句话都实实在在，尽量把各项工作转化为具体的项目，体现在工作落实上，不论对政府工作的要求，还是对政府自身建设的要求，都讲求实效，务求实效。

　　二、6个小故事意味深长，对闽北转变观念的启示

　　前两年我解读政府工作报告时，主要是对原文内容的深化。现在，大家对许多过去陌生的概念，如沿江快速通道、高速铁路等等，都已经比较清楚了，并成为大家的常识。所以，今天我不想再对报告内容作过多的解释，而是想通过6个小故事，对报告派生的一些概念作闸述，以此启发大家的思路。前几天，龚市长在北京全国“两会”期间，接受了福建日报采访，认为南平要实现跃升发展的既定目标，必须解决三个问题，一是观念转变，二是时空转换，三是科学发展。对于时空转换问题，我市通过大量的现代立体交通设施建设，正在逐步改善；对于科学发展，市委、市政府即将开展深入学习实践科学发展观活动。我在这里想讲一讲转变观念问题，主要是通过6个小故事进行闸述，如有不对之处敬请批评指正。

　　1、等车的启示：抢抓发展机遇

　　等车的故事说：有一个乡下农民在城里，肩挑背扛，带的东西很多，要坐公交车回家。当他到车站等车时，旁边等车的人很多。有人对他说，你带的东西多，等一下上车；有人说，我家很远，我要先上车；有人说，你再等一等，坐下一部豪华大巴车。其实，这是一辆末班车，结果这位老实巴交的农民当然没上车，只能在外面过夜。

　　这个故事说明：闽北长期以来有一种不急、不争、不抢的小农经济意识。过去，一直说要克服等、靠、要的思想。等、靠是不对，但要还是要的。闽北人更为可悲的是，等、靠、让，结果让出了机遇，让出了发展，让出了宝贵的时间。时间是一维性，时不我予，时不可待，时不再来。从文化背景来分析，农耕文化、小农经济意识在山区干部根深蒂固。农耕文化有其好的一面，勤俭、淳朴，有超稳定的结构。如“三十亩地一头牛，老婆孩子热炕头”，东北人就是典型的代表，“小酒喝喝，小曲唱唱”、“过了山海关，人人都是赵本山”。也有不好的一面，与市场经济快节奏、激烈竞争的要求格格不入。在现代市场经济下，如果还是按部就班，就会丧失很多发展机遇。如福银高速和向莆快速铁路，就是因为我们争取不够，丧失了历史发展机遇。

　　这个故事告诉我们：要克服等、靠、让的传统观念，增强急、争、抢的闯拼意识。急的是责任和效率，争的是政策和项目，抢的是机遇和发展。因此，必须解放思想、树立信心、抢抓机遇、加快发展，这是今年市“两会”的主题，也是今后一个时期的重点工作。

　　在急、争、抢方面，我们也有一些成功的例子。去年上半年争取了京福高速铁路。这条高铁按时速350公里的客运专线标准设计，总投资260亿元，南平境内200公里左右。从京沪高速铁路的安徽蚌埠站引出，经合肥、黄山、江西上饶至我省武夷山、南平、福州，是贯穿我国南北的第一条高速铁路大通道。项目建成后，从北京到福州只要7个多小时，武夷山到福州约50分钟，南平到福州约半小时。高速铁路是现代立体交通的核心，机场、铁路、公路都是它的配套，还会带来经济的发展、生活观念的变化。如日本新干线带动了东京湾周边几个县的发展，台湾新干线也带动了台南和台中的发展。

　　当前，闽北有许多历史性机遇。今年政府工作报告中分析了我们面临的有利机遇，一是我市经济外向依存度相对较低，有利于在应对国际金融危机中趋利避害，减少负面影响；二是中央和省上出台的保增长、扩内需、调结构、重民生的政策措施，有利于我市推动产业转型升级、完善基础设施、改善民生；三是我市是农业大市，强农惠农的政策导向，有利于夯实“三农”基础，发展现代农业，推动农村改革发展；四是我市正处于重大基础设施建设高峰期，有利于增强投资拉动和工业支撑，形成新的经济增长点。因此，我们学会捕捉和抢抓发展机遇，主动对接，敢于作为，最大限度把政策转化为项目，转化为实实在在的成果。

　　2、光棍娶妻的启示：把握发展的阶段性

　　光棍娶妻的故事说：一个光棍，家里没一口锅、一张床，就连寡妇都不愿意嫁给他。他只有把生活条件改善了，才可能娶到媳妇。

　　这个故事的启示：一个地方或一个单位，在发展过程中必须把握发展的阶段性。要加快发展，必须从基础抓起，必须明确现阶段的工作目标。不然，发展必然是事倍功半。

　　对南平来说，交通条件、区域优势、华侨的人缘优势比沿海发达地区都欠缺。现在要跃升发展，必须从基础做起，必须做足“通”的文章。一是加大招商引资。我们在浦南高速公路通车后，才开展大规模的招商，争取在本届政府任期内引进1000家以上的规模以上企业。反之，如果我们在条件不具备条件进行招商，只会取得事倍功半的效果。二是发展武夷山会展经济。去年我们为举办茶博会建设了许多基础设施，特别是建成标准展馆，具备了发展会展经济的条件。今年就吸引安踏、七匹狼等大型企业来武夷山召开订货会，有力地带动了旅游产业的发展。三是正确认识投资、出口、消费三驾马车拉动问题。如果一个地方没有优质的产品出口，谈何出口拉动，如果老百姓没有富起来，谈何消费拉动。作为闽北，我们当前要实行政府投资为主体的投资拉动，之后才会有工业经济拉动、消费拉动。四是注重统筹协调。统筹资源，政府要统筹高速公路、土地、矿产、木材资源；统筹分配，把有限的资金更多地用于民生保障；统筹人的全面发展，在财力有限的情况下，在众多的社会事业中，要优先发展教育，提高全民素质。

　　3、买打火机的启示：树立现代融资理念

　　买打火机的故事说：近几年，南平进入基础建设高峰期，需要大量的资金投入。如十条高速公路要投资600亿，七条铁路要投资500亿，还有沿江快速通道、汽车服务中心为代表的城建项目也需要投资100亿。张兆民副市长对高速公路投资有一个形象的比喻，说高速公路建设像一座大山，要炸掉这座大山，买炸药需要600亿元的资金，南平需要200亿元的投入买雷管，其中政府需要60亿元的资本金买导火线，分摊到每年需要10－20亿元用来买打火机。资金怎么筹措呢？把所有公用资产统一起来交给国投公司融资，所得资金用于土地收储，收储的土地向银行抵押贷款，用于资本金。如此几轮循环后，就可以解决资本金问题。

　　这个故事告诉我们：要树立现代融资理念。农耕社会最怕的是负债，市场经济最需要的是融资。搞企业需要融资，政府也需要融资。政府通过合理融资建设基础设施，既使城市升值，也改善发展条件。融资的评判标准不是单纯看欠银行多少钱，而是看资产质量的高低，关键是要通过合理融资把资产做优做大，既降低负债率，提高资产质量，又能改善生产生活条件。

　　树立现代融资理念的核心是资源、资产、资金的运作和统筹，如何把资源变成资产，把资产变成资金。要以激活市场为目标，积极构建灵活多样的融资体系。我市成功组建邮政储蓄银行和建瓯石狮村镇银行，加快筹建农村合作银行，金融产业初现雏形。深化政府与金融机构的战略合作，市政府与农发行、开发银行光大银行、民生银行签订了金融合作协议。整合全市国有资产，做大做强国投公司、城建国投公司、交通国投公司三大融资平台，进一步破解重大项目建设融资难题。今后，如果政府要控制战略性资源，就可以通过招拍挂由这三大公司购买；再如政府的办公大楼也可以交由这些公司运作，各个单位和部门有偿使用办公设施，从而提高公司的现金流量，将资产变成资金。

　　4、“首都之争”的启示：建旧促新，建设组团式山水园林城市

　　南平的行政中心曾两次在南平和建阳间迁移，搬一个扔一个，导致两个城市都没发展好。省委对南平提出“一点一线一重心”战略后，更引发了“首都之争”。对此，我认为要建旧促新。谋划建设新兴发展区域并不是摒弃老城区，而是新老互相促进、共赢发展。只有把老城区做好，才有基础和实力去推动新区发展。

　　未来的城市规模是“两心多组团”。我们可以有两个中心，一个是现有的南平中心城市，要按照组团式山水园林城市的定位，做好第一批、第二批48项城建重点项目，加快推进沿江快速通道和景观通道建设，做大做强做精做美中心城市；另一个是新兴发展区域，该区域位于建阳、武夷山之间，面积600平方公里，数条高速公路及铁路、机场贯穿其中。它的北面是武夷山城区，西北面是武夷山景区和度假区，南面是闽北产业集中区，基础设施条件非常完善，具备了建设一个现代城市的所有功能。目前，我们主要是打好新兴中心区域的基础设施基础、产业基础和城市规划基础，统筹考虑高速铁路与重大基础设施的对接，以及新兴发展区域规划、主体功能区规划、土地利用总体规划、耕地保护区规划、风景区规划的统一，做到有机统一、高度统一。

　　行政中心迁移往往是城市建设中一大妙招，可以借此实现经营城市的新突破。古今中外均有类似的例子。一是巴西为了扭转本国南北贫富差别悬殊的局面，甚至采取了迁都的方式，于1960年4月将首都正式从1300多万人口的里约热内瓦，迁到巴西利亚，带来了明显的效果。巴西利亚原是一座仅有10多万人的小城，工农业均不发达，现在已成为拥有10个卫星城、面积5814平方公里，人口达200万的现代化都市。首都的迁移还使巴西中部地区这片不毛之地的农牧业生产得到迅速发展，工商业初具规模，经济面貌发生翻天覆地的变化。二是我国的青岛市。上世纪90年代初，青岛市政府做出了非常果断的决策，把位于老城的政府机关所在地卖掉，在新城建立了新的行政区。这项举措同时实现了两个效果，一个是通过空间结构的转换，带动整个城市资产的转换，原来的行政办公机构大部分位于城市最好的地段，这里的土地是非常值钱的。但政府占在这个地方不产生经济效益，而如果政府把这个地方交给企业，会使整个城市创造新的效益。政府拿了这笔钱之后在新区建设一个规模更大、更漂亮、或环境更好的行政办公区，周围地价会升值，很多新的公共设施就会围绕新区发展起来，便带来第二效益。三是深圳市。实际上改革开放以来深圳的行政中心就一直在漂移，至今已经迁了3次，第一次是在原来的宝安区，后来又从罗湖迁到上步，上步同样飞速发展起来，非常繁荣，现在深圳又迁到了福田区。整个城市也沿着这个轨迹不断向外延，取得了令世人惊奇的成就。

　　5、两个箩筐的启示：把发展中心村作为新农村建设的突破口

　　新农村建设由来已久，梁漱溟在30年代提出新农村建设，毛泽东在文革期间曾开展社会主义新农村建设。进入新世纪以来，中央再次提出新农村建设。但是，以前的新农村建设和现在的新农村建设是两个不同的箩筐，市委、市政府提出的中心村建设与一些干部思维定式中的农民居民点建设，也是两个不同的箩筐。

　　中心村的概念：以区域位置和经济发展条件较好的农村居民点为中心，聚集周围一些自然村和散居村民，形成具有一定规模和良好生产、生活环境，并对周边一定区域的经济、社会发展起辐射作用的新型乡村聚落。中心村要选择在适宜人居、交通便利、功能齐全的地方建设。

　　为什么要建中心村？闽北有2万多个自然村，我们在公共产品和社会服务上不可能村村覆盖。特别在经济转型期间，很多农民出去打工，老人要照顾，小孩要上学，更需要我们建中心村，让公共资源集中到一个地方，让更多的农民能享受到公共服务，提高公共产品对人口的动态覆盖率。

　　中心村要做好四个规划：一是经济发展规划。重在因地制宜，宜工则工、宜农则农、宜商则商，发展主导产业，壮大产业规模，统筹产业布局和生态环境。二是村镇建设规划。重在合理配置土地资源，根据近郊、中郊、远郊及高山、平原等不同区域的特点，统筹生产、居住、社会事业和农村基础设施建设。三是劳动就业规划。重在提高农民素质，转移农村劳动力，增加务工收入。四是基层组织建设规划。重在增强农村干部队伍活力，提高民主管理水平，使村级组织建设有方向、有目标，能落实、能发展。

　　我个人认为中心村应具备以下几条标准：一是经济发展。中心村镇的经济总量和农民人均收入增幅高于所在县（市、区）的平均水平，主导产业明晰，有产业特色的村建有专业合作经济组织；二是设施改善。中心村镇的路网、电网、自来水网、广播电视网、通讯信息网、垃圾污水处理网健全完善。三是公共服务。中心村镇的教育有保障，适龄儿童就近入学，九年义务教育后的初中毕业生绝大多数能进入普通高中或中等职业教育学习；卫生有场所，有一个卫生医疗所，村民小病实现就近医治，有一个卫生公厕，建立村容卫生保洁制度；养老有保障，有一所敬老院，建立与经济发展水平相适应的农村养老保险制度，不断扩大新型农村合作医疗制度和农村最低生活保障制度覆盖范围；娱乐有质量，有一个文体活动中心，定期开期娱乐活动。四是乡风文明。村规民约健全完善，村民养成良好的公民道德素质和卫生习惯，自觉爱护公共设施，保持环境卫生整洁，维护社会治安稳定。五是管理规范。村建规划落实到位，“六大员”发挥作用，村务管理民主，村级组织健全有力。

　　6、“木头烂在山上就是文化”的启示：整合提升闽北优势特色产业

　　前年，龚市长在武夷山调研时，在景区里看到一颗几百年的树倒下，认为不能将木头锯掉取走，而要在上面架一个梯子，把木头保护起来，形成我们保护生态的文化。对此，武夷山在生态保护和发展工业上有非常清晰的认识，主动调减木材采伐指标，今年基本不砍阔叶林，并节燃改材，保护生态，涵养水源。

　　南平有地球上同纬度最好的生态环境，青山绿水就是闽北最大的资源、最大优势、最大支撑，是海西绿色腹地战略定位的基础。所以我们要在保护生态的基础上，突出发展生态主导型的产业，促进经济发展与人口、资源、环境相协调。一定要保护好我们的生态。致力于生态示范区的建设。今后进一步建设生态市。生态示范区更多的保护的问题，生态市是在保护的基础上发展。

　　现代区域经济的形态已从农耕主导型、产业主导型、城市主导型、信息主导型，发展到生态主导型。因此，我们要加快培育新兴产业，尽快把旅游、生物、养生、创意产业及矿产资源的深度加工等产业做强做大。

　　三、学习政府工作报告，重在加强学习、解放思想，更新观念

　　一要勤于学习，善于学习。我们事业关键在党，关键在人。只有把充分调动人的主观能动性，才能形成推动发展的力量。广大党员干部要认真学习理论，深刻领会科学发展观内涵，努力学习现代经济知识、金融知识，学习科学技术知识和社会管理知识。要善于学习先进发达地区的经验和教训，学习他们的打拼精神，而不是结果。

　　二要打破定式，打破惯性。很多同志看问题，只看到一个点、一条线，或一个平面，没能从历史、空间、发展、联系、立体的角度看问题。如到外地学习参观时，有的同志看了很激动，回来没行动。因此，要讲究思维的维度问题，提高思维的维度、丰度、精度、开放度，开拓思路，提升思维能力。

　　三要胸襟开放，从政开明。在大开放年代，闽北如何跃升发展，一定要以宽阔的胸襟、开放的思维，承接一切美好的东西。前不久，省委副书记于广洲在南平调研时，提出科学发展就是开放发展，大开放才有大思路。从于副书记的讲话中，我们可以理解，要用大开放，行大智慧，谋大发展。比如，我认为不作为就是最大的不廉政，不作为比不出事更可怕。不能只艰苦不奋斗，我们要奋斗不艰苦，这样的奋斗才有意义。

　　四要注重运作，注重实践。我们都担任一定的领导职务，掌握一定的权力，必须考虑如何解放思想、转变观念、科学发展、跃升发展。发展是无上限，实践也是无上限的。因此，我们要注重运作、注重实践。
